# Burden of road traffic injuries in Federation of Bosnia and Herzegovina – fifteen-year survey: Šeila Cilović Lagarija

**DOI:** 10.1093/eurpub/ckac131.578

**Published:** 2022-10-25

**Authors:** Š Cilović-Lagarija, S Skočibušić, S Musa, A Jogunčić

**Affiliations:** 1 Institute of Public Health for FBiH, Sarajevo, Bosnia and Herzegovina; 2 Public Health Institute of Canton Sarajevo, Sarajevo, Bosnia and Herzegovina

## Abstract

**Introduction:**

Road traffic injuries (RTI) are among the ten leading causes of death worldwide, and they are the leading cause of death among young adults aged 15-29 years. In Federation of Bosnia and Herzegovina (FBiH), with population of 2,1 million, around 380 people die every year and additional 11.000 get seriously injured in RTI.

**Aim:**

The aim of this study was to investigate the incidence and fatality rate of RTI in FBiH in the period of 2006 until the end of 2020.

**Methods:**

Data source of RTI (ICD-X) incidence and fatality rate was Statistical book for Federation of Bosnia and Herzegovina, which includes all injuries and deaths reported through Ministry of internal affairs. Fatality rate was calculated as number of confirmed deaths in total number of reported RTI. To compare frequencies of reported deaths Chi square test was used. YLD were calculated based on information on injury that was caused in road traffic accidents.

**Results:**

In the last 15 years, in FBiH, total 39,455 injuries related to road traffic were reported. According to the official data, over the period 2006-2020 the peak of fatality rate (8.52% deaths among all cases with RTI) was in 2016, while it had a statistically significant decline in 2018: 6.51%; 2019: 6.35%; and 2020: 6.32% (x2=7728,584; p < 0,0001). It is estimated that young adults (in the age group 30 to 39 years) injured in the road traffic accidents have 197.01 patient-years of total 881.17 years to live with disabilities just based on serious RTI in 2020.

**Conclusions:**

RTI pose a significant burden on the health of the population in FBiH. After implementing strict laws in the year 2017, and 2018, a significant decrease of RTI was registered, including the number of deaths due to RTI (fatality rate).

This abstract is support and sponsorship by ‘BoCO-19 - The Burden of Disease due to COVID-19'. Project is coordinated/led by Robert Koch Institute and supported by the WHO Regional Office for Europe.

**Key messages:**

• RTI are significant burden for health of population in FBH. Strict laws and stronger punishments and fees are decreasing number of RTI.

• This abstract is support and sponsorship by ‘BoCO-19 - The Burden of Disease due to COVID-19'. Project is coordinated/led by Robert Koch Institute and supported by the WHO Regional Office for Europe.

